# A Comparative Study of Brachial–Ankle Pulse Wave Velocity and Heart–Finger Pulse Wave Velocity in Korean Adults

**DOI:** 10.3390/s20072073

**Published:** 2020-04-07

**Authors:** Jaegeol Cho, Hyun Jae Baek

**Affiliations:** Department of Medical and Mechatronics Engineering, Soonchunhyang University, Asan, Chungnam 31538, Korea; jaegeolcho@sch.ac.kr

**Keywords:** PWV (pulse wave velocity), baPWV (brachial–ankle PWV), photoplethysmography (PPG)

## Abstract

Arterial stiffness is considered an index of vascular aging. The brachial–ankle pulse wave velocity (baPWV) method is widely used because of its proven effectiveness; and the pulse wave velocity measurement method using both electrocardiogram (ECG) and photoplethysmogram (PPG) is actively being studied due to the convenience of measurement and the possibility of miniaturization. The aim of this study was to evaluate and compare the effects of age and gender in Korean adults using both the baPWV method and the PWV method with ECG and finger PPG (heart–finger PWV). The measurements have been carried out for 185 healthy subjects of Korean adults, and the results showed that the baPWV was highly correlated with age in both genders (r = 0.94 for both males and females). However, the correlation values in heart–finger PWV measurement were significantly lower than those of baPWV (r = 0.37 for males and r = 0.71 for females). Although the heart–finger PWV method is suitable for mobile applications because it can be easily miniaturized while maintaining its signal quality, these results show that the heart–finger PWV method is not as effective as baPWV at evaluating the arterial stiffness.

## 1. Introduction

One of the major causes of death worldwide is cardiovascular disease, especially in developed countries, and cardiovascular morbidity and mortality are known to be related with increased arterial stiffness, which raises blood pressure. Aortic stiffness, which is the stiffness of the largest vessel and the most important clinically, is an independent predictor of cardiovascular mortality and fatal stroke in patients with essential hypertension [1,2]. Moreover, aortic pulse wave velocity, which reflects the aortic stiffness, has been shown to be an independent predictor of coronary heart disease and stroke, even in apparently healthy subjects in a large-population-based study [3]. 

In previous studies, several terms describing the mechanical properties of arteries were used, such as arterial stiffness, compliance, and distensibility. For a given pressure change in a blood vessel, compliance and distensibility refer to the dimensional change and fractional change in volume or cross-sectional area, respectively. On the other hand, arterial stiffness is a descriptive term that cannot be quantified by compliance and distensibility [4]. To quantify the arterial stiffness, compliance, distensibility, and Young’s modulus can be used; however, the values of these terms are not readily available by non-invasive methods. Therefore, several other methods such as second-derived photoplethysmography, carotid-femoral pulse wave velocity, and forearm reactive hyperemia were used to assess arterial stiffness, and the carotid-femoral pulse wave velocity (PWV) method was found to be a more effective predictor of cardiovascular events in hypertensive patients [5]. A number of studies have also demonstrated that measurement of aortic PWV is the best available non-invasive measure of arterial stiffness, and it correlates well with subsequent risk for cardiovascular diseases [6].

In general, aortic PWV can be measured with commercial devices using tonometry. A pressure-sensing probe is located on carotid and femoral arteries and the pressure waveforms are recorded for the calculation of the time difference between the two measuring sites. The aortic PWV or carotid-femoral PWV (cfPWV) can be obtained by dividing the measured distance between the two separated sites with the time difference. The time difference can be determined two ways. One is to measure consecutive pressure waveforms coupled with electrocardiography at two sites, and the time differences between the characteristic points of ECG and pressure waveforms generate the relative time difference between the two measuring sites. The other way is to record pressure waveforms at two different sites simultaneously for direct calculation of time difference. Since the accuracy of carotid-femoral PWV is limited by technical difficulty of the measurement [6], brachial–ankle pule wave velocity (baPWV) calculated from volume-rendering waveforms using blood pressure measurement cuffs in extremities has been considered as a surrogate of cfPWV, which has been proved as a marker reflecting aortic PWV by previous studies [7,8]. 

The usefulness of baPWV can be found in several studies. It can be used as a predictive marker for cardiovascular events, especially ischemic stroke risk [9] and a predictor of mortality in elderly Chinese on the conditions of markedly increased PWV and hypertension [10]. Measurement of baPWV during the acute phase of stroke can be useful for identifying patients at high risk of mortality [11], and is associated with the severity and presence of coronary artery disease [12]. Recent studies have shown that an elevated baPWV level increases cardiovascular risk, providing additional predictive information for future cardiovascular disease [13], and may be a useful independent predictor of incident hypertension [14].

Brachial–ankle PWV using cuffs at extremities can be automatically measured [15,16], and its accuracy was proven as high as the manual measurement method [16]. Therefore, there have been several studies to find the relationship between baPWV and vascular aging. Tomiyama et al. [17] evaluated the influences of age and gender in 12,517 subjects of Japanese, adults and Miyai et al. [18] obtained the data on baPWV from 3215 Japanese adolescents ranging from 12 to 18 years and developed the baPWV distribution curves by age and gender. Recently, Yiming et al. [19] established the reference and normal values of baPWV in a Central Asian population.

PWV measured at two sites can be applied to various parts of arteries of body as well as the aorta. The PWV measurement using ECG and PPG also can be applied to several parts of the arteries [20]. It can be used to assess the stiffness of the arterial portion from the heart to the PPG measuring site. Using a finger, toe, or earlobe, the volumetric waveforms can be easily detected by measuring the transmission of infrared light through skin. The measurement of the PWV is, ideally, measured using pressure sensors or ultrasonic probes at two sites, but since the peaks in three waveforms of ECG, PPG, and pressure pulse show very good agreement [21], PWV can be obtained by combining two of ECG, PPG, pressure pulse, and blood flow waveforms. The main advantage of PWV measurements using ECG and PPG is that they can measure both hands in a non-supine position, and there are several commercially available analog front-end (AFE) chips for ECG and PPG measurements [22], making it possible to miniaturize the measuring device. 

In particular, ECG and PPG signals are widely used for the evaluation of cardiovascular function. Characteristics of PPG waveforms such as amplitude, peak-peak time, and dicrotic notch were used for blood pressure estimation [23,24,25,26,27], hypertension assessment [28], and cardiovascular risk evaluation [29]. Since noise-free signals are very important when using the feature points of a biosignal, silicon photomultipliers (SiPMs) were adopted instead of the conventional photodiode (PD) [30] to obtain clear PPG waveforms. In addition, there have been several approaches to remove the distortion and artifacts of ECG and PPG signals using various techniques, such as pattern recognition [31] and neural networks [32]. Recently, improved sensing elements and signal processing techniques have been used for estimating blood pressure with increased accuracy [27].

The purpose of this study was to evaluate and compare the effects of aging and gender with two types of PWV method, baPWV and heart–finger PWV (the heart–finger PWV means the PWV measured using ECG and finger PPG, and this notation is used in the remainder of this paper), for healthy Korean adults. Another aim of this study was to compare and verify the values of heart–finger PWV measured with a small mobile device with those of the commercial apparatus used in hospitals.

## 2. Materials and Methods

**Figure 1:**
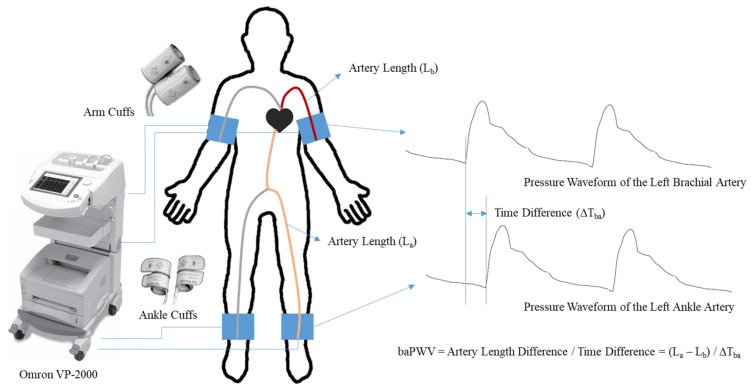
Measurements of brachial–ankle pulse wave velocity (PWV). The time difference between the brachial and ankle pressure waves is used to calculate brachial–ankle PWV (baPWV).

The brachial–ankle PWV was measured using an automated device (VP-2000, Omron, Japan), which provided both left side and right side baPWV measured from the cuffs wrapped on both upper arms and ankles. The subjects were examined in supine position for baPWV measurement and the measurements were repeated twice consecutively. Figure 1 shows a detailed description of brachial–ankle PWV measurement. As is shown in the figure, the device simultaneously measures oscillometric waveforms and calculates the time intervals between the characteristic points (peak, valley, maximum slope, etc.) of the brachial waveform and the corresponding peaks of the ankle waveform. The distances between the measuring sites of baPWV were calculated automatically according to the heights of the subjects. As a result, two sets of baPWV values representing the left and right side of baPWV were recorded for each subject. 

**Figure 2:**
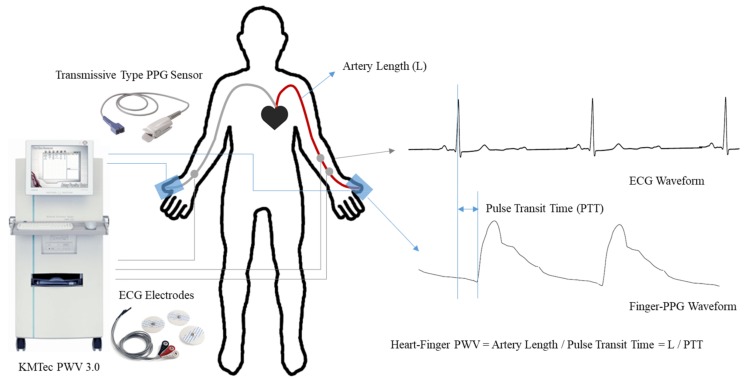
Measurements of heart–finger PWV. The time difference between the characteristic points of ECG and PPG waveforms is pulse transit time (PTT) and is used to calculate heart–finger PWV.

The heart–finger PWV was also measured with an automated device (PWV 3.0, KMTec, South Korea), which has three electrodes for ECG measurements and four transmissive type optical sensors for PPG measurements of the fingers and toes. Figure 2 shows a detailed experimental setup for heart–finger PWV measurement. The subjects were in a seated position for heart–finger PWV measurements, and the measurements were repeated three times consecutively. For ECG measurements, two electrodes were attached on the left lower arm and one electrode was attached on the right lower arm. Two finger clip type PPG sensors were located on both left and right thumbs, and both hands were covered to prevent the noise of light. The measurement takes 30 s and provides two values of pulse transit time (PTT); one is for the left arm and the other is for the right arm. As is shown in Figure 2, the pulse transit time is often defined as the time interval between the ECG R peak and the onset of PPG pulse. Because PWV is defined as the distance travelled divided by PTT, the heart–finger PWV was calculated by measuring the distances from the heart to both thumbs along the body surface using a tape measure. Thus, two heart–finger PWVs were recorded for each subject with two sets of PTT and arm length in a single measurement.

To compare the baPWV and heart–finger PWV, a total of 185 healthy Korean adults (92 males and 93 females) were recruited by Samsung Medical Center (Seoul, Korea) with IRB approval (IRB No. 200709052). The age of subjects recruited ranged from 20 to 66. The “healthy subjects” were defined as non-smoking people who had no history or symptoms of cardiovascular disease and a body mass index (BMI) of less than 25. The healthy subjects were also screened through hematological, hemochemical, and urine tests at the first visit, and the measurements of baPWV and heart–finger PWV were made at the second visit. Therefore, the PWV data set presented in this study represent the normal range of PWV values for “healthy Korean adults” with no history or symptoms of cardiovascular disease. The blood pressures of the subjects were measured with the auscultatory method by skilled nurses. The mean blood pressure (MBP) was calculated as MBP = DBP + (SBP – DBP)/3, where SBP and DBP are mean systolic blood pressure and diastolic blood pressure, respectively. 

**Figure 3:**
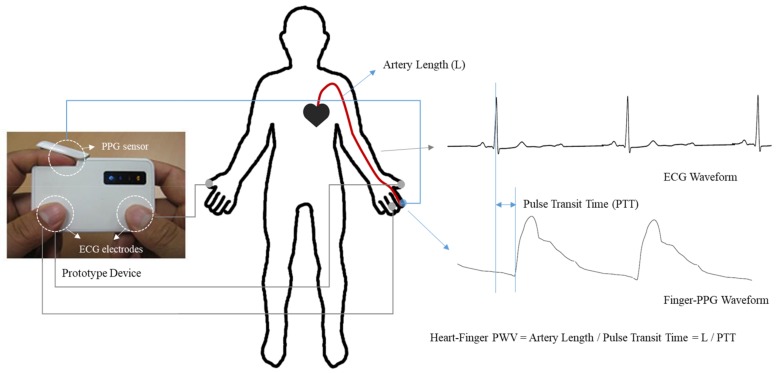
Measurements of heart–finger PWV with a prototype device equipped with contact-type ECG electrodes and a transmissive type PPG sensor.

To attempt a mobile heart–finger PWV measuring device, a small prototype device was made using commercial AFE (analog front-end) chips for ECG and PPG. Figure 3 shows the measurement method and photograph of the PWV measuring device which has a transmissive type PPG sensor and contact-type ECG electrodes. The PPG sensor is composed of an LED of 940 nm wavelength and a Si PIN photodiode (KDP6004A, Kodenshi AUK, 4.0 × 5.4 mm^2^, spectral sensitivity 700–1100 nm), which are widely used for commercial and clinical devices, such as pulse oximeters. Besides the clinical study at hospital, ten subjects were recruited additionally, and heart–finger pulse transit time were measured both with the prototype device and the commercial equipment for hospital use (PWV 3.0). For each subject, a total of twelve measurements were made (three on the left arm and three on the right arm per device). The PWV measurements were performed by alternating the measuring device to minimize the effect of time variation. Since the purpose of the experiment was to compare the PWV values of the two different devices in the same person, arm length measurements were skipped and the PTT values were compared instead of the PWV values.

## 3. Results and Discussion

### 3.1. Brachial–Ankle PWV and Heart–Finger PWV Measurements

The results are expressed as means ± standard deviations. Statistical analysis was performed to find the Pearson correlation coefficient r and p-values, and a value of p < 0.05 was considered statistically meaningful. Table 1 shows the characteristics of all subjects who participated in the clinical study. The mean values of systolic blood pressure, diastolic blood pressure, brachial–ankle PWV, and heart-ankle PWV of females were lower than those of males, which is consistent with previous studies [17,18].

Table 2 depicts the anthropometrics of all 185 participants (92 males and 93 females) classified in chronological order. For direct comparison with the previous results of Tomiyama et al. [17], the subjects were grouped at 5-year age intervals according to their age. The mean and standard deviation of the subgroup data were used as representative values in the plot. As mentioned above, the baPWV measuring device automatically calculated the artery length from the height and recorded the PWV values. Since the baPWV values were obtained on both the left and right sides, it was necessary to determine whether two values should be used separately for one subject or only the average could be used. 

For this purpose, we tested whether the baPWV data classified by gender could be regarded as normally distributed, but the baPWV data was not. Therefore, we compared the baPWV data of the left and right sides using the Mann–Whitney U test and found that the two baPWV dataset could be regarded equal (p = 0.657 for males, p = 0.472 for females, significance level = 0.05). In addition, since the correlation coefficients between the left and right side baPWV data were found to be very high (r = 0.96, p < 0.00001 for males and r = 0.97, p < 0.00001 for females), only the average value of baPWV was recorded for one participant finally.

Since the heart–finger PWV equipment provided pulse transit time, not the PWV, the PWV values of the left arm and the right arm were calculated by measuring the artery lengths in the left arm and the right arm, which are shown in Table 2. The heart–finger PWV values of both left and right arms could be regarded as normal distributions, so the inequalities of the two groups were tested using a two-sample t-test, and we considered that there was no difference between both arms in both males and females (p = 0.75 for males and p = 0.73 for females, significance level = 0.05). In this case as well, the PWV values measured in both arms were very similar (r = 0.94, p <0.00001 for males and r = 0.93, p <0.00001 for females); the mean values of both arms were used as representative values.

**Figure 4:**
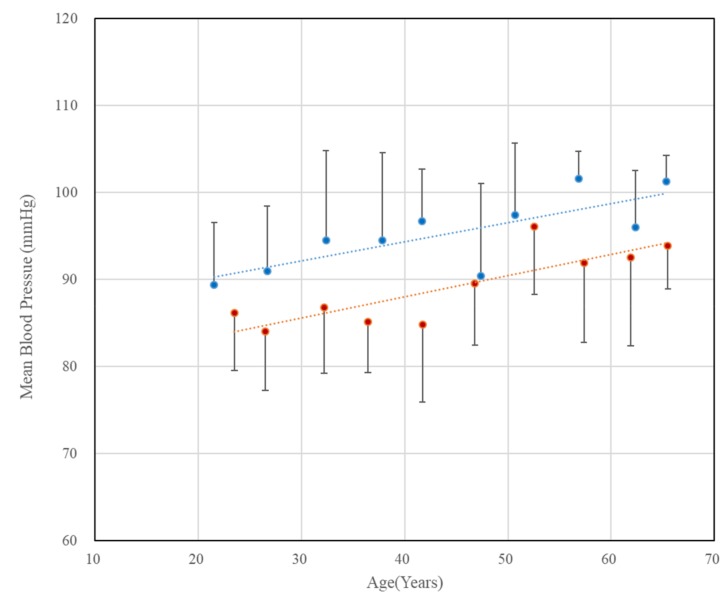
Chronological changes in mean blood pressure in both genders. The error bars represent the standard deviations (blue: males, r = 0.77, p = 0.009, red: females, r = 0.84, p = 0.002).

Figure 4 shows the mean blood pressure changes for men and women, respectively. The error bars in the figure represent the standard deviations of each data point. For the clarity of the figure, only the upper part of standard deviation for men and the lower part of standard deviation for women are shown. As shown in the figure, mean blood pressure significantly increased in both males and females with age, and mean blood pressure of females was lower than that of males in the same age group. This result was consistent with previous study [17], although the number of subjects decreased.

**Figure 5:**
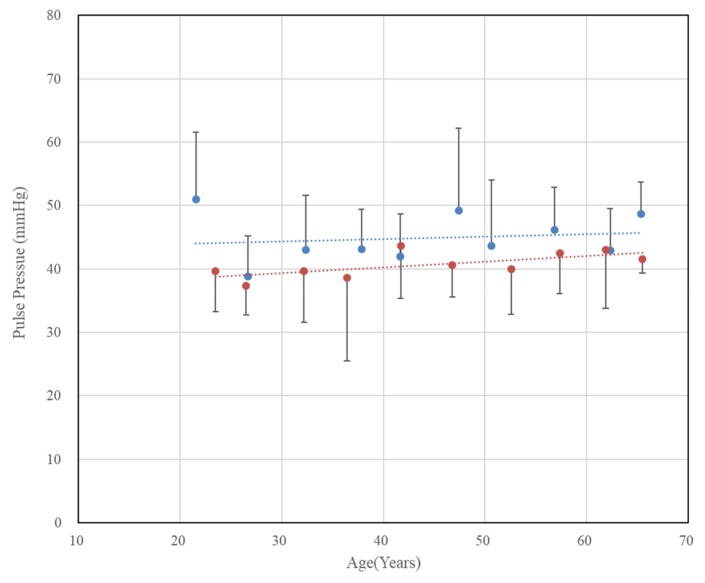
Chronological changes in pulse pressure (the difference between systolic blood pressure and diastolic blood pressure) in both genders. The error bars represent the standard deviations (blue: males, r = 0.147, p = 0.685, red: females, r = 0.663, p = 0.036).

Unlike the mean blood pressure, the pulse pressure shown in Figure 5 does not show a significant change with age. In the case of females, there was a tendency to increase with age (p < 0.05), but in males, there was no significant trend change according to age. Although it is generally known that arterial stiffness increases with age, previous study [17] did not show a significant correlation between pulse pressure and age for males. Thus, it is expected that the pulse pressure change of males does not match well with age.

**Figure 6:**
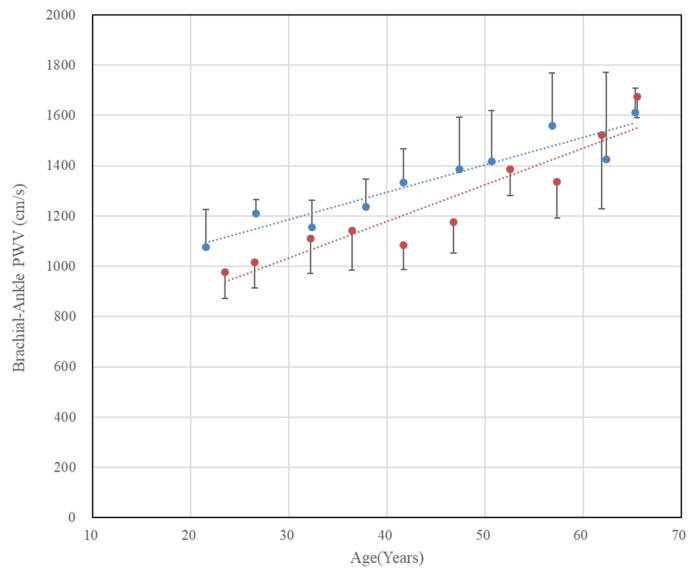
Chronological changes in brachial–ankle pulse wave velocity (baPWV) in both genders. The error bars represent the standard deviations (blue: males, r = 0.94, p < 0.0001, red: females, r = 0.94, p < 0.0001).

Figure 6 shows the change in baPWV with age. Although there exist differences in the magnitude of the standard deviations by age, both males and females show a marked increase trend according to age. The linear relationships between age and baPWV by gender have been derived by regression analysis as follows.

Male:baPWV=10.93×age+857.2 (r=0.94, p<0.0001)

Female:baPWV=14.56×age+595.6 (r=0.94, p<0.0001)

As can be seen in Figure 6, women (red dot) have a lower baPWV than men (blue dot) of the same age group, but the difference between men and women decreases with increasing age. This is consistent with the results of a previous study [17], wherein women’s baPWV was lower than men’s, and the difference gradually decreased with age.

**Figure 7:**
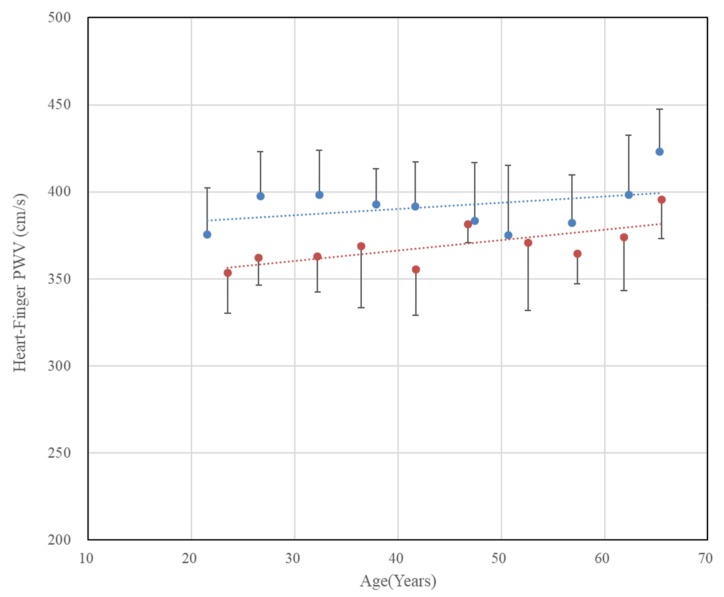
Chronological changes in heart–finger pulse wave velocity in both genders. The error bars represent the standard deviations (blue: males, r = 0.37, p = 0.286, red: females, r = 0.71, p = 0.021).

In the case of the heart–finger PWV shown in Figure 7, the tendency is quite different from that of baPWV. First, the increase in heart–finger PWV with age was not clear in both males and females. A gradual but significant increase was observed in females (r = 0.71, p < 0.05); however, in males, the correlation coefficient was not high (r = 0.37), and the correlation was not significant either (p = 0.286). These results suggest that heart–finger PWV is not effective at measuring arterial stiffness according to age. There could be two main reasons for these results.

The one reason is that the aging of the heart–finger artery (the portion of the artery from the heart to the point of finger PPG measurement) is different from the aging of the aorta. In a previous study comparing 20 patients with coronary artery disease and 24 healthy subjects [15], the carotid-femoral PWV and baPWV of CAD patients were 71% and 55% higher than those of the healthy controls, respectively, whereas heart-brachial PWV were only 14% higher in CAD patients compared to healthy subjects. The other reason is that the use of ECG R-peaks reduced the accuracy of the heart–finger PWV method. In fact, there is a time difference which is called pre-ejection period (PEP) between the time of R peak occurrence (ventricle depolarization) and the time occurrence of actual blood ejection from the ventricle. Payne et al. [33] confirmed that the tendency of pre-ejection period to change largely because of drugs, and recently, Balmer et al. [34] showed that there was a difference between the two values of PWV in pigs, one of which was measured with ECG and a pressure catheter, and the other was measured with two pressure catheters in the same arteries. Therefore, if PEP varies due to various conditions, such as the individual deviation, drugs, and so on, the pulse transit time will vary according to the factors other than arterial stiffness, which will be expected to degrade the predictive accuracy of heart–finger PWV as a diagnostic tool of atherosclerosis.

**Figure 8:**
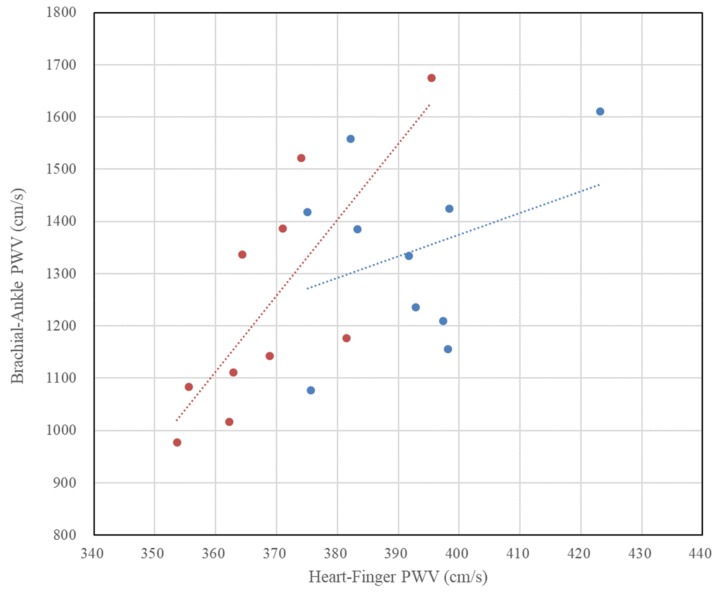
Relationship between brachial–ankle pulse wave velocity and heart–finger pulse wave velocity (blue: males, r = 0.34, p = 0.336, red: females, r = 0.79, p = 0.006).

**Figure 9:**
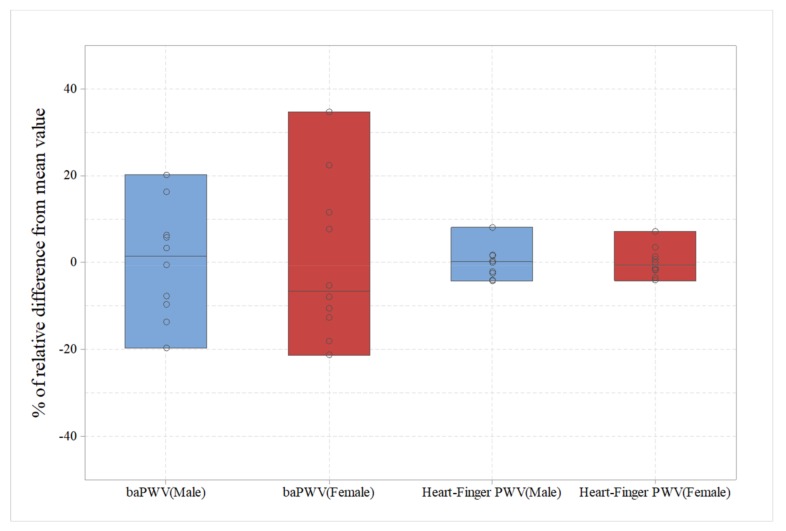
Relative data ranges according to PWV measurement methods and genders.

Figure 8 compares the baPWV and heart–finger PWV measurements. As expected, a relatively high correlation (r = 0.79, p <0.01) was shown in female data, but a significant relationship between baPWV and heart–finger PWV was not found in male case. Figure 9 shows the ranges of change in the values of baPWV and heart–finger PWV according to age. In the figure, the y-axis represents the difference from the mean value in percent. In the case of baPWV, 20%–30% of the mean value changes with age, but heart–finger PWV changes within ± 10%. If PWV is considered as a sensor or sensing method for measuring atherosclerosis, the sensitivity of heart–finger PWV sensor is about 20%–30% of baPWV.

As a result of this study, the heart–finger PWV method using the ECG sensor and the PPG sensor is very simple and easy to measure because it can be measured with the subject in a sitting position, using both hands. However, it was confirmed that this method is not as effective as baPWV in the measurement of the evaluation of aging in arteries. It might be useful to utilize heart–finger PWV as a tool for the management of arterial health, which needs more clinical evidence beyond the range of this study. Approaches to improve the effectiveness of heart–finger PWV are still ongoing, such as a recent study [35], wherein various features extracted from ECG and PPG signals besides the PTT were utilized and combined with machine learning algorithms. Additionally, local PWV measurement [36] could be an alternative to arterial health assessment, since heart–finger PWV measures the average stiffness of the arteries from heart to finger.

### 3.2. Comparison of Heart–Finger PWV Measurements

**Figure 10:**
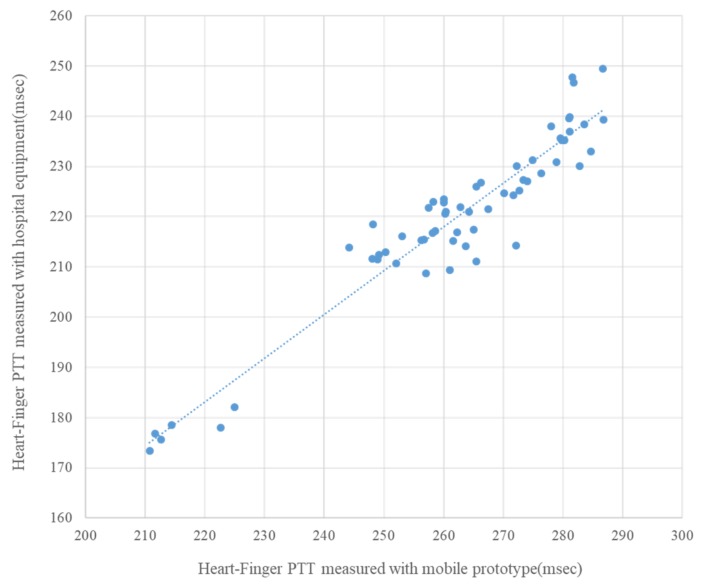
Comparison of pulse transit time data measured with commercial hospital equipment and a mobile prototype in 10 subjects (r = 0.95, p < 0.0001).

Figure 10 compares the heart–finger pulse transit time (PTT) values measured with the hospital equipment (PWV 3.0, Figure 2) used in the comparative clinical study of baPWV and heart–finger PWV with the mobile device shown in Figure 3. As shown in the figure, the measurement results of the hospital equipment and the mobile device prototype showed a very high correlation (r = 0.95). It can be seen that the pulse transit time measured by the two devices differs by several tens of msec, which can be changed depending on which characteristic point is selected when calculating the time difference from the peak of the ECG waveform and the PPG waveform. For example, the time difference can be measured between the R peak of ECG and the peak of PPG waveform or between the R peak of ECG and the waveform of 1st derivative of PPG. Therefore, a shift of several tens of msec may occur depending on the method of calculating the time difference.

The contact type ECG electrodes and PPG sensors of mobile devices have the advantage that they can be measured in everyday life, since they can be built in small devices, such as smart phones and smart watches. As demonstrated in this study, small-sized mobile devices can measure the heart–finger PWV at the same level as hospital equipment. Therefore, it has the advantage of being able to measure and manage from time to time, if the usefulness of heart–finger PWV can be verified.

## 4. Conclusions

Arterial stiffness can be assessed by PWV measurement, which is relatively simple and non-invasive. PWV is related to the elasticity modulus of an artery and gradually increases with age. In the present study, brachial–ankle PWV and heart–finger PWV measurements were performed on 185 Korean adults without symptoms or history of cardiovascular disease. Through the experiments, the effects of age and gender have been assessed, and the results of the two methods have been compared. From the baPWV results, which were classified at 5-year age intervals, linear regressions to age in Korean adults were obtained with very high correlations in both genders. However, the correlation values in heart–finger PWV measurements were significantly lower than those of baPWV measurements. The use of the ECG R peak, which is considered the starting point of the pulse transit, can cause a deviation due to the pre-ejection period, and atherosclerosis between the heart and finger shows a different aging tendency from baPWV. We also found that the heart–finger PWV method is simple and can be miniaturized maintaining its signal quality; however, the heart–finger PWV method is not as effective as baPWV at evaluating the arterial stiffness, especially the aortic stiffness which is known to reflect atherosclerosis. 

## Figures and Tables

**Table 1:** Characteristics of all subjects (n=185). (BMI: body mass index, SBP: systolic blood pressure, DBP: diastolic blood pressure, MBP: mean blood pressure, PP: pulse pressure, baPWV: brachial–ankle pulse wave velocity).

	Male	Female
Number	92	93
Age	42 ± 13	42 ± 13
Height (cm)	172 ± 7	159 ± 5
BMI (kg/m^2^)	23 ± 2	22 ± 2
SBP (mmHg)	124 ± 11	116 ± 10
DBP (mmHg)	80 ± 8	75 ± 8
MBP (mmHg)	95 ± 8	88 ± 8
PP (mmHg)	44 ± 8	41 ± 8
Right Arm Length (cm)	85 ± 4	78 ± 3
Left Arm Length (cm)	84 ± 4	77 ± 3
Brachial–Ankle PWV (cm/s)	1297 ± 230	1195 ± 231
Heart-Finger PWV (cm/s)	391 ± 29	365 ± 25

**Table 2:** Anthropometrics of subjects (n = 185) in chronological classification (RA: right arm, LA: left arm).

Gender	Age (Year)	No. of Subjects	Height (cm)	BMI (kg/m^2^)	SBP (mmHg)	DBP (mmHg)	RA Length (cm)	LA Length (cm)
Male	20–24	13	177 ± 7	22 ± 1	123 ± 11	72 ± 8	87 ± 4	87 ± 4
	25–29	6	175 ± 6	24 ± 1	117 ± 9	78 ± 7	88 ± 4	87 ± 4
	30–34	11	176 ± 6	23 ± 1	123 ± 14	80 ± 9	87 ± 4	86 ± 4
	35–39	14	173 ± 5	23 ± 2	123 ± 13	80 ± 9	85 ± 3	85 ± 3
	40–44	16	171 ± 4	23 ± 1	125 ± 8	83 ± 6	83 ± 3	83 ± 3
	45–49	5	172 ± 7	22 ± 2	123 ± 10	74 ± 13	85 ± 3	84 ± 4
	50–54	6	167 ± 4	23 ± 1	127 ± 12	83 ± 8	82 ± 1	82 ± 2
	55–59	6	165 ± 9	24 ± 1	132 ± 6	86 ± 4	82 ± 5	81 ± 4
	60–64	12	165 ± 4	22 ± 2	125 ± 10	81 ± 6	82 ± 2	81 ± 2
	65–69	3	169 ± 10	23 ± 1	134 ± 3	85 ± 4	84 ± 4	84 ± 3
Female	20–24	10	162 ± 4	21 ± 1	113 ± 7	73 ± 8	79 ± 3	79 ± 3
	25–29	10	160 ± 3	21 ± 2	109 ± 8	72 ± 7	78 ± 2	78 ± 2
	30–34	15	160 ± 3	21 ± 1	113 ± 9	74 ± 8	78 ± 2	77 ± 2
	35–39	7	161 ± 6	22 ± 2	111 ± 7	72 ± 9	78 ± 3	78 ± 3
	40–44	14	160 ± 6	21 ± 2	114 ± 11	70 ± 9	78 ± 4	78 ± 4
	45–49	5	161 ± 5	21 ± 2	117 ± 9	76 ± 7	78 ± 2	78 ± 2
	50–54	7	157 ± 3	22 ± 1	123 ± 12	83 ± 6	78 ± 3	77 ± 3
	55–59	14	157 ± 5	22 ± 1	120 ± 11	78 ± 9	77 ± 3	77 ± 3
	60–64	9	155 ± 5	23 ± 1	121 ± 15	78 ± 8	76 ± 3	76 ± 3
	65–69	2	155 ± 4	23 ± 2	122 ± 4	80 ± 6	78 ± 4	78 ± 2
